# Sex-Specific Differences in Left Ventricular Mass and Volumes with Body Mass Index among Children Aged 6 to 8: A Cross-Sectional Study in China

**DOI:** 10.3390/nu15133066

**Published:** 2023-07-07

**Authors:** Huidi Xiao, Wen Shu, Menglong Li, Liyuan Xu, Nubiya Amaerjiang, Jiawulan Zunong, Sten H. Vermund, Dayong Huang, Mei Chong, Yifei Hu

**Affiliations:** 1 Department of Child, Adolescent Health and Maternal Care, School of Public Health, Capital Medical University, Beijing 100069, China; huidi_x@mail.ccmu.edu.cn (H.X.); shuwen@student.pumc.edu.cn (W.S.); menglong.li@mail.ccmu.edu.cn (M.L.); xxxinury@mail.ccmu.edu.cn (N.A.); jiawulanzn@ccmu.edu.cn (J.Z.); 2 Department of Growth and Development, Capital Institute of Pediatrics, Beijing 100020, China; 3 Peking Union Medical College, Chinese Academy of Medical Sciences, Beijing 100730, China; 4 Department of Echocardiography, Beijing Anzhen Hospital, Capital Medical University, Beijing 100029, China; xuliyuanyx@163.com; 5 Yale School of Public Health, Yale University, New Haven, CT 06510-3201, USA; sten.vermund@yale.edu; 6 Department of Hematology, Beijing Friendship Hospital, Capital Medical University, Beijing 100050, China; hdayong@ccmu.edu.cn

**Keywords:** obesity, sex, left ventricular mass, left ventricular structure, children, cohort

## Abstract

Few studies have examined the sex differences in left ventricle (LV) structure and physiology from early life stages. We aimed to assess the role of sex and overweight/obesity on left ventricular mass (LVM) and LV volume in Chinese children without preexisting cardiovascular risk factors. We selected 934 healthy children aged 6–8 years from an existing cohort in Beijing, China. Linear regression models were used to regress body mass index (BMI), fat mass, systolic blood pressure, diastolic blood pressure, waist circumference, and visceral fat area (VFA) with LVM, left ventricle end-diastolic volume (LVEDV) and end-systolic volume (LVESV). Higher BMI, fat mass, waist circumference, VFA, and stroke volume (SV) predicted higher LVM, LVEDV, and LVESV in both sexes. Multivariable analysis showed that boys with an elevated BMI had greater LV hypertrophy. LVEDV and LVESV were higher among boys than among girls and increased with higher BMI in both boys and girls. LVEDV and LVESV were associated with VFA in boys. We observed sex differences in LVM, LVESV, and LVEDV among prepubertal children, independent of obesity, with higher values observed in boys. Sex differences in cardiac structure in children may help explain the higher incidence of cardiovascular disease in male adults. Whether interventions to reduce childhood obesity can improve the trajectory of cardiac dynamics is worth investigating.

## 1. Introduction

The prevalence of obesity among children and adolescents is increasing worldwide, contributing to the increasing threat of adult cardiovascular disease (CVD) [1]. A limited pool of literature on higher body mass index (BMI) (i.e., overweight and obesity), waist circumference, and fat levels suggests that childhood status may influence adult CVD risk [2,3,4]. Physiological differences between the sexes may contribute to CVD risk differences in obese children [5] and, perhaps, in children with a lower BMI (i.e., normal weight) as well.

Studies have confirmed that increased left ventricular mass (LVM) can lead to left ventricular hypertrophy (LVH) among adults [6]. A retrospective cross-sectional study among children and adolescents aged 2–19 years found that increased LVM could be attributed, in part, to increased BMI and that the male sex was an independent predictor of elevated LVM [7]. LVH and left ventricular (LV) remodeling are major predictors of CVD [8], while increased left ventricular end-diastolic volume (LVEDV) is a key indicator of negative LV remodeling [9]. Based on the Framingham Heart Study, male sex and higher BMI at baseline were risk factors for abnormal heart structure during follow-up and a lower probability of recovery from abnormal LV geometry [10]. In contrast, females who had structural abnormalities and a lower BMI at baseline were more likely to return to a normal LV geometric structure during follow-up [10]. Studies have consistently shown that LVM and heart structures differ between the sexes among obese adults.

Previous studies on obesity-induced LV structural changes have largely focused on adults, and many have included participants with obesity-related complications, such as diabetes and hypertension that are known independent modifiers of LVM. Only a few studies have examined sex differences from the early life stage, emphasizing the role of obesity in the modification of LVM and LV structure in men, women, or combined groups. This study aimed to examine the potential sex differences in LV structure and physiology in children aged 6–8 years. M-mode and 2-dimensional (2D) echocardiography were utilized to investigate whether sex differences are noted in children, both with and without obesity, and how they may impact LVM and LV volume size in the absence of preexisting cardiovascular risk factors.

## 2. Materials and Methods

### 2.1. Study Design and Participants

Beijing Children Growth and Health Cohort (“PROC”, hereafter referred to as the cohort) was established to study risk factors of obesity and CVD along the childhood growth continuum, to identify possible interventions to reduce CVD risks. The participants are planned to be followed until adulthood in Shunyi District, Beijing, China. We approached parents or guardians of 2394 children aged 6–8 years old in six public non-boarding schools. Children with mental illnesses and/or congenital heart and/or lung diseases were excluded. All the participating children and their parents signed informed consent forms. We conducted anthropometric measurements and Echocardiography measurements, collected blood and urine samples, and assayed routine and biochemical hematuria indicators in 1914 children (80%) who agreed to participate from October 2018 to June 2019. The study protocol and informed consent were reviewed and approved by the Ethics Committee of Capital Medical University (No. 2018SY82). The study protocol was registered in China Clinical Trial Registry (https://www.chictr.org.cn/index.html (accessed on 10 May 2023), No. ChiCTR2100044027).

### 2.2. Inclusion Criteria

All the children were screened for the presence of identifiable risk factors of CVD. Children were eligible for the study if they had no known cardiovascular risk factors, such as congenital heart/lung diseases, history of any other cardiovascular dysfunction, hypertension, high glucose (fasting glucose ≥ 5.6 mmol/L) [11], or high total cholesterol (total cholesterol ≥ 5.17 mmol/L) [12]. All eligible children were normotensive at the time of inclusion in this study. Based on the above inclusion criteria, 934 children out of 1914 were eligible and participated in this study.

### 2.3. Laboratory Assays and Anthropometric Measurements

Laboratory assays and anthropometric measurements were conducted at the sequential baseline survey. (1) Blood serum biochemical assay: After a 10 h fasting period, we collected 10 mL of venous blood from the children and analyzed total serum cholesterol and blood glucose using a US AU5800^®^ automatic biochemical analyzer (Beckman Coulter, Inc., Shizuoka, Japan). (2) Body composition analysis and weight: We measured bioelectrical impedance with an H-Key350 body composition analyzer (Beijing Seehigher Technology Co., Ltd., Beijing, China) to assess participants’ body composition and weight. The child was instructed to stand barefoot on the pad of the analyzer, wearing only light clothing, after a 10 h fast and post-micturition. (3) Height: We used a mechanical height meter for standing height, taking the average of two measurements. (4) Waist circumference: We used a soft band ruler (validated with a steel ruler) to measure the horizontal girth through the center of the umbilicus, or the girth of the midpoint line between the lowest point of the rib and the upper edge of the iliac crest. We averaged the two waist circumference measurements as the final value. (5) BMI: We used International Obesity Task Force (IOTF) guidelines to calculate BMI [13]. (6) Blood pressure: We conducted three consecutive blood pressure measurements and averaged the last two readings as the final value, using the OMRON HBP-1300 blood pressure monitor (OMRON HBP-1300, Dalian, China). We applied the 2016 European Society of Hypertension guidelines for the management of high blood pressure in children and adolescents as the blood pressure standard [14].

### 2.4. Echocardiography Measurement

We performed M-mode and 2D echocardiographic imaging (2D/M ECHO) on all children from January to April 2019 with an Aplio 500 Platinum Series ultrasound (Canon Medical Systems Inc., Tochigi, Japan) with a probe frequency of 2.5~4 MHz, according to the measurement method recommended by the American Association of Echocardiography. The 2D/M ECHO allowed us to quantify various parameters of LV volume and function, including LVM, LVEDV, left ventricular end-systolic volume (LVESV), LVM-indexed height, and LVM-indexed height^2.7^. A trained physician measured the left ventricular end-diastolic diameter (LVEDD), left ventricular end-systolic diameter (LVESD), left ventricular end-diastolic posterior wall thickness (LVPWT), and interventricular septal thickness (IVST) over 3 cardiac cycles and the average values were used for analysis. To ensure that all children were free of overt cardiovascular disease and that left ventricular geometry was not altered due to heart failure or cardiomyopathy, left ventricular ejection fraction (LVEF%) was also recorded. Using the formula recommended by Devereux [15], we calculated the LVM:

LVM = 0.80 × [1.04 × (IVST + LVPWT + LVEDD)^3^ − LVEDD^3^] + 0.6 g (1)

LVEDV and LVESV were calculated based on the LVEDD and the LVESD using the Teichholtz correction formula, which is an M-mode ultrasound volume calculation method [16]:

V = 7.0/(2.4 + D) × D^3^ (2)

We used two formulas to calculate the LVM index:

LVM-indexed height = LVM/height(3)

LVM-indexed height^2.7^ = LVM/height^2.7^ (4)

Stroke volume (SV) was calculated as:

SV = LVEDV − LVESV(5)

### 2.5. Statistical Analysis

We used SAS^®^ 9.4 software (SAS Institute, Inc., Cary, NC, USA) for data analysis. All normally distributed data were presented as the mean ± standard deviation, while non-normally distributed data were presented as the median and interquartile range (P_25_–P_75_). We used analysis of variance (ANOVA) with Bonferroni correction for normally distributed data and the Kruskal–Wallis test for non-normally distributed data. Participants were grouped according to sex and the IOTF guideline categories. Using Pearson correlation analysis, we examined the relationship between independent variables (BMI, fat mass, SV, waist circumference, and VFA) and outcome indicators (LVM, LVEDV, LVESV, LVM-indexed height, and LVM-indexed height^2.7^). Univariate linear regression was used to compare LV systolic function between boys and girls. After testing for collinearity, we retained the variables with a variance inflation factor < 5. We used multiple linear regression analysis to assess the effects of BMI on LVM, LVEDV, LVESV, LVM-indexed height, and LVM-indexed height^2.7^. Two-tailed *p* < 0.05 was considered statistically significant.

## 3. Results

### 3.1. Blood Pressure and Left Ventricular (LV) Function

Age and diastolic blood pressure (DBP) were similar between boys and girls in the normal, overweight, or obese groups (Table 1). All participants exhibited normal LVEF%, without any notable differences between boys and girls in any BMI group (Table 1). Notably, there was no substantial correlation between BMI and LVEF% in either boys (r = 0.04, *p* > 0.05) or girls (r = 0.06, *p* > 0.05).

### 3.2. Sex Differences in LV Hypertrophy and Volume Size Considering Obesity

#### 3.2.1. LV Hypertrophy and Volume Size in Boys

**Figure 1:**
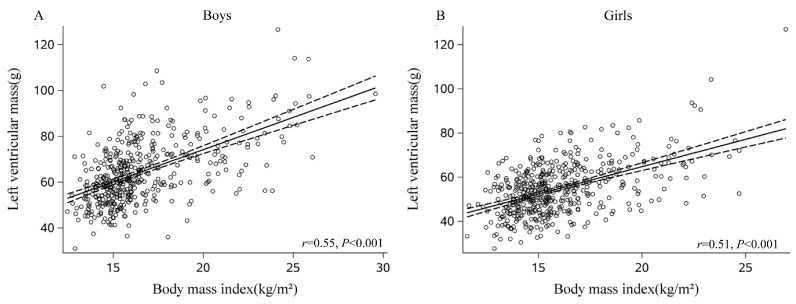
Sex-specific correlations between body mass index (BMI) and left ventricular mass (LVM) (**A**, boys; **B**, girls) showed a steeper relationship between BMI and LVM in boys. Mean ± 95% CI (dashed lines) are shown for each graph.

**Figure 2:**
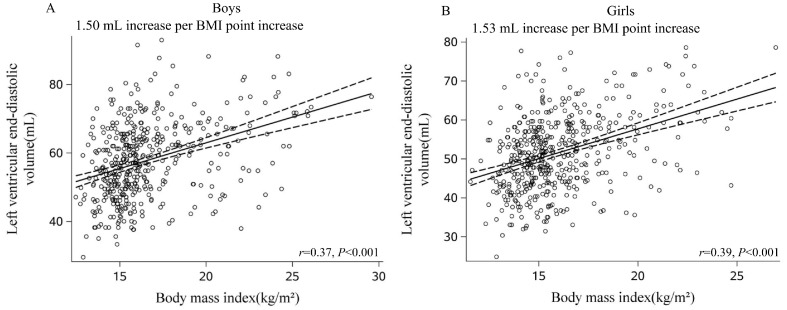
Sex-specific correlations between body mass index (BMI) and left ventricular end-diastolic volume (LVEDV) (**A**, boys; **B**, girls) showed a steeper relationship between BMI and LVEDV in girls. Mean ± 95% CI (dashed lines) are shown for each graph.

**Figure 3:**
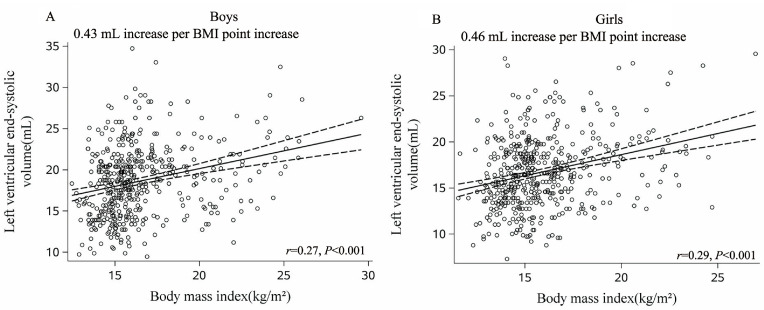
Sex-specific correlations between body mass index (BMI) and left ventricular end-systolic volume (LVESV) (**A**, boys; **B**, girls) showed a steeper relationship between BMI and LVESV in girls. Mean ± 95% CI (dashed lines) are shown for each graph.

Overweight boys had greater LVM than normal-weight boys (71.6 ± 13.8 g vs. 61.3 ± 11.8 g, *p* < 0.05) and had less LVM than obese boys (80.1 ± 15.8 g, *p* < 0.05, Table 1). BMI (r = 0.55), fat mass (r = 0.54), waist circumference (r = 0.53), VFA (r = 0.45), and SV (r = 0.69) were associated with LVM in boys (all *p* < 0.001; Figure 1A and Table S1). In terms of LVM-indexed height and LVM-indexed height^2.7^, we observed similar associations (Figures S1A and S2A). LVEDV and LVESV were similar between overweight and obese boys (*p* > 0.05, Table 1). LVEDV and LVESV were different between normal versus overweight boys and between normal versus obese boys. Higher BMI, fat mass, waist circumference, VFA, and SV were correlated with increased LVEDV and LVESV in boys (Figure 2A and Figure 3A, Table S1). Aggregate data suggest that boys exhibit LV cavity dilatation associated with increased body fat and elevated LVM.

#### 3.2.2. LV Hypertrophy and Volume Size in Girls

Overweight girls had greater LVM than normal-weight girls (60.9 ± 11.3 g vs. 52.5 ± 9.8 g, *p* < 0.05). Moreover, obese girls had a greater LVM than overweight and normal-weight girls (both *p* < 0.05, Table 1). As seen among boys, BMI (r = 0.51), fat mass (r = 0.51), waist circumference (r = 0.52), VFA (r = 0.45), and SV (r = 0.69) were associated with LVM in girls (Pearson correlation analyses, all *p* < 0.001, Figure 1B and Table S1). Furthermore, these variables were also associated with LVM-indexed height and LVM-indexed height^2.7^ (both *p* < 0.001, Figures S1B and S2B). Similar to boys, LVEDV and LVESV did not differ between overweight and obese girls (Table 1). There were significant differences between LVEDV and LVESV among normal versus overweight girls as well as among normal versus obese girls. Higher BMI, fat mass, waist circumference, VFA, and SV were associated with elevated LVEDV and LVESV in girls (Figure 2B and Figure 3B, Table S1). The data suggested that girls, similar to boys, presented with dilated heart cavities along with increased body fat and LVM.

### 3.3. Comparison of Sex-Specific Hypertrophy and Volume Size in Obesity

#### 3.3.1. Left Ventricular Mass (LVM)

According to univariate regression analysis between BMI and LVM in boys and girls, boys showed a greater LV hypertrophic response to elevated BMI (boys: +2.82 g vs. girls: +2.47 g per 1 kg/m^2^ increase in BMI, *p* < 0.001, Table S1). Moreover, LVM was also positively correlated with age, systolic blood pressure (SBP), fat mass, waist circumference, and VFA (Table S2). Similarly, LVM-indexed height was positively associated with BMI, SBP, fat mass, waist circumference, and VFA. LVM-indexed height^2.7^ was not associated with SBP but was positively associated with almost all anthropometric indicators of obesity, e.g., BMI, fat mass, waist circumference, and VFA (Table S2). Multiple regression analysis showed that LVM was positively associated with BMI (adjusted for age), SBP, DBP, and VFA in both boys and girls. Notably, the steeper slope of association between LVM and BMI among boys compared to girls was still observed even after controlling for other factors in multivariable analysis (boys: +2.94 g vs. girls: +2.16 g per 1 kg/m^2^ increase in BMI, *p* < 0.001, Table 2).

#### 3.3.2. LV End-Diastolic Volume (LVEDV)

The LVEDV increased with BMI in both boys and girls, and the increase was greater in girls than in boys (boys: +1.50 mL vs. girls: +1.53 mL per 1 kg/m^2^ increase in BMI, both *p* < 0.001, Table S1). This again suggested that LV hypertrophy in boys and girls due to obesity may be associated with heart cavity expansion. Slightly different from LVM, LVEDV was not related to age but was positively associated with SBP, fat mass, waist circumference, and VFA (Table S2). Similarly, after adjusting for age, SBP, DBP, and VFA, the LVEDV was associated positively with BMI both for boys and girls (*β* = 2.29 for boys vs. *β* = 1.49 for girls, *p* < 0.001). In addition, LVEDV was associated with VFA only in boys (*p* < 0.05) but not in girls (*p* > 0.05). This suggests that VFA can only be used to predict higher LVEDV among boys (Table 2).

#### 3.3.3. LV End-Systolic Volume (LVESV)

The LVESV increased more with elevated BMI among girls than boys (boys: +0.43 mL vs. girls: +0.46 mL per 1 kg/m^2^ increase in BMI, *p* < 0.001, Table S1). Similarly, LVESV was positively associated with fat mass, waist circumference, and VFA (Table S2). After adjusting for age, SBP, DBP, and VFA, the LVESV was positively associated with BMI both for boys and girls (*β* = 0.70 for boys vs. *β* = 0.41 for girls, *p* < 0.001). In addition, LVESV was associated with the VFA in boys (*p* < 0.05) but not in girls (*p* > 0.05) (Table 2).

## 4. Discussion

Previous studies have suggested that obesity-related LV adaptation and remodeling may originate in childhood, with LV hypertrophy as an adaption to the enlargement of the LV cavity and impairment of diastolic function [17]. The relationship between obesity and LVM and LV geometry can be predicted by BMI or fat mass [18,19]. Among children without apparent cardiovascular risk factors, our study found that obese boys showed greater LV hypertrophy compared to obese girls. LVESV and LVEDV increased with increasing BMI in both boys and girls, while only related to VFA in boys. Considering the sex disparities in LVM and chamber size [20,21], our findings suggest potentially important differences in cardiovascular risks between boys and girls in response to obesity.

LV hypertrophy and obesity in children and adolescents have been extensively studied, showing a strong association between cardiac geometric parameters and obesity [9,22,23]. A study on ethnic and sex differences in coronary risk development in young adults, utilizing 2D speckle tracking echocardiography (STE), reported obesity to be associated with LV hypertrophy, with males exhibiting higher LVM than females [4]. Another retrospective clinical cohort study reported that male sex and BMI jointly predicted LVM, with BMI as an independent LVM predictor, i.e., sex differences in LV hypertrophy associated with obesity [3]. However, the authors acknowledged a limitation in their study, namely the lack of data on the prevalence of metabolic syndrome among the participants, which could potentially influence the accuracy of the results regarding LV hypertrophy associated with obesity. Therefore, it is crucial to consider cardiovascular risk factors such as hypertension, diabetes, hypercholesterolemia, and smoking history to accurately disentangle the role of sex differences in the interplay of obesity and LV structure and function.

To our knowledge, our study has an unprecedented sample of healthy children to study the role of childhood obesity in the remodeling of LV geometry. Our relatively large study demonstrated a link between obesity and LV hypertrophy even among healthy children. Obesity had a significant effect on LVM, independent of hypertension, diabetes, and high cholesterol, as children with these risk factors were excluded from our study. These findings align with another study that used cardiovascular magnetic resonance imaging to study the relationship between obesity and LVM in healthy adults. That study reported LV hypertrophy among obese individuals, which was influenced by sex-related disparities and remained independent of variables such as blood pressure, age, and diabetes [24].

A British birth cohort study found that higher BMI and overweight were associated with higher LVEDV and LVM in both childhood and adolescence. Furthermore, it was observed that longer durations of overweight in childhood and adolescence were associated with a rise in LVEDV [25]. The increase in LVEDV, attributed to cumulative exposure to overweight, can be interpreted as an indicator of increased preload, consistent with the evidence suggesting that obese individuals require increased preload reserve. Our study also found that LVEDV and LVESV in boys were associated with VFA, but this phenomenon was not observed in girls. Additionally, LVESV in girls was associated with BMI. Whether this is a transient physiological phenomenon or a persistent discrepancy between males and females remains unknown. Sex differences may play a role in influencing LVM, SV, and cardiac function in varying degrees during childhood and adolescence. We are currently investigating this aspect in our ongoing PROC cohort study, as visceral fat is recognized as a significant risk factor for metabolic syndrome.

Among various fat storage pools in the human body, visceral adipose tissue has been strongly implicated in linking obesity to cardiometabolic disease [26]. It is well known that regular endurance exercise not only reduces visceral adipose tissue volume but also significantly decreases the overall risk of cardiometabolic diseases [27,28,29]. A Korean adult cohort study reported that men have higher VFA compared to women [30]. Additionally, a 10-week study of progressively vigorous-intensity interval training among older adults with abdominal obesity showed that exercise had a positive effect on the reduction of VFA in men [31]. Therefore, it is crucial to remain vigilant regarding the increase in VFA in boys and implement vigorous physical activity interventions to mitigate the risk of cardiovascular diseases.

The present study has several notable strengths. Firstly, it involved a large community sample of healthy children aged 6–8 years old across a wide range of BMI categories, allowing for the examination of associations between metabolically healthy obesity [32] and measures of left ventricular mass (LVM) and chamber size. Importantly, the study focused on children without other cardiovascular risk factors, ensuring that the observed associations were specifically related to obesity. This definition of metabolically healthy obesity [32], which excludes metabolic disorders and cardiovascular diseases such as type 2 diabetes, dyslipidemia, hypertension, and atherosclerotic cardiovascular disease in individuals with obesity, provides valuable insights into the early predictors of cardiovascular risk. Moreover, our cohort study preceded the onset of puberty in all children, allowing for the examination of early predictors of CVD risk and minimizing potential confounding from hormonal changes associated with puberty. The sub-population included in this study was a representative sample of the entire cohort, enhancing the generalizability of the findings to the broader population of children. Furthermore, we used several indicators of obesity measurements in addition to BMI, such as VFA, enabling us to triangulate our main research outcome: sex discrepancy in heart structure, adjusted for BMI. Lastly, we employed 2D/M ECHO, a well-validated and reliable method, to measure the outcomes of interest since it has been recommended for large-scale epidemiological studies to measure the effects of childhood obesity on LVM and chamber size.

We acknowledge some limitations in this study. A lack of geographic diversity (children all from Beijing) limits the generalizability of the findings. Furthermore, the ECHO data were cross-sectional, so we could not study the dynamic changes in the relationship between obesity and chamber size. However, we will continue to assess other indicators for future target organ damage, including renal, cholesterol, liver function, hearing loss, and others, through the ongoing cohort, offering longitudinal observations of the relationship between sex and LV structure in Chinese children in the context of BMI.

## 5. Conclusions

Sex discrepancies in LV chamber size were observed in children aged 6–8 years, before the onset of puberty, independent of obesity and in the absence of other cardiovascular risk factors. Boys exhibited larger LVM, LVESV, and LVEDV compared to girls. Additionally, in boys, LVESV and LVEDV were associated with the visceral fat area (VFA), highlighting the importance of not overlooking boys when implementing vigorous physical activity interventions. Given the younger age of participants, it is plausible to assume that early intervention to reverse childhood obesity may potentially alter the trajectory of increasing LVM and chamber size. The sex-related differences in LVM and chamber size, adjusted for obesity, observed in children could help explain the higher incidence of cardiovascular disease (CVD) in males during adulthood.

## Figures and Tables

**Table 1:** Anthropometric and left ventricular characteristics of children aged 6–8 grouped by IOTF BMI categories.

Variable	Normal Weight		Overweight		Obese	
	Boys (*n* = 357)	Girls (*n* = 389)	Boys (*n* = 51)	Girls (*n* = 57)	Boys (*n* = 47)	Girls (*n* = 33)
Age (years)	7.1 ± 0.3	7.1 ± 0.3	7.1 ± 0.3	7.1 ± 0.3	7.2 ± 0.3	7.1 ± 0.3
BMI (kg/m^2^)	15.4 ± 1.1 *^,†,‡^	15.0 ± 1.2 ^§,||^	19.1 ± 0.8 **	18.8 ± 0.8 ^††^	23.0 ± 1.9	22.3 ± 1.5
SBP (mmHg)	100.6 ± 7.2 *^,‡^	98.2 ± 7.1 ^§^	100.7 ± 7.9 **	101.1 ± 8.2	105.5 ± 7.0 ^‡‡^	99.4 ± 7.4
DBP (mmHg)	55.2 ± 5.7	55.1 ± 5.4 ^§^	56.6 ± 5.6	57.2 ± 5.6	55.8 ± 6.1	55.8 ± 6.8
Waist circumference (cm)	54.1 ± 3.7 *^,†,‡^	52.4 ± 3.6 ^§,||^	64.7 ± 4.2 ^¶,^**	62.9 ± 4.3 ^††^	74.8 ± 6.3 ^‡‡^	71.5 ± 5.9
VFA (cm^2^)	16.3 (13.4–19.9) *^,†,‡^	17.9 (14.8–21.6) ^§,||^	35.0 (25.1–47.3) **	35.4 (28.7–43.3) ^††^	77.5 (57.7–87.1)	72.4 (63.1–84.7)
LVEF% (%)	67.4 ± 4.3	67.3 ± 4.1	68.2 ± 4.4	67.5 ± 4.4	68.2 ± 3.6	68.0 ± 4.5
LVEDV (mL)	56.1 ± 10.5 *^,†,‡^	50.2 ± 8.9 ^§,||^	62.9 ± 10.0 ^¶^	55.6 ± 9.5	64.8 ± 12.2	60.4 ± 10.8
LVESV (mL)	18.2 ± 4.3 *^,†,‡^	16.4 ± 3.6 ^§,||^	19.9 ± 3.7 ^¶^	18.1 ± 4.0	20.4 ± 4.5	19.2 ± 4.2
LVM (g)	61.3 ± 11.8 *^,†,‡^	52.5 ± 9.8 ^§, ||^	71.6 ± 13.8 ^¶,^**	60.9 ± 11.3 ^††^	80.1 ± 15.8 ^‡‡^	70.4 ± 16.3
LVM-indexed height (g/m)	49.0 ± 8.8 *,^†,‡^	42.4 ± 7.4 ^§,||^	55.9 ± 10.6 ^¶,^**	47.9 ± 8.2 ^††^	60.9 ± 11.0 ^‡‡^	54.2 ± 11.3
LVM-indexed height^2.7^ (g/m^2.7^)	33.5 ± 5.9 *^,†,‡^	29.5 ± 5.1 ^§,||^	36.8 ± 7.5 ^¶^	31.9 ± 5.2 ^††^	38.3 ± 6.6 ^‡‡^	34.9 ± 6.5
Total cholesterol (mmol/L)	4.3 (3.9–4.7)	4.4 (3.9–4.7)	4.1 (3.6–4.6)	4.3 (4.0–4.6)	4.5 (4.0–4.8)	4.0 (3.7–4.8)
Fasting glucose (mmol/L)	5.1 (4.8–5.3) *	5.0 (4.7–5.2)	5.1 (4.8–5.4) ^¶^	5.0 (4.8–5.2)	5.2 (5.0–5.3) ^‡‡^	5.0 (4.9–5.1)

BMI: body mass index; SBP: systolic blood pressure; DBP: diastolic blood pressure; VFA: visceral fat area; LVEF%: left ventricular ejection fraction; LVEDV: left ventricular end-diastolic volume; LVESV: left ventricular end-systolic volume; LVM: left ventricular mass. * *p <* 0.05, normal-weight boys vs. normal-weight girls; ^†^ *p <* 0.05, normal-weight boys vs. overweight boys; ^‡^ *p <* 0.05, normal-weight boys vs. obese boys; ^§^ *p <* 0.05, normal-weight girls vs. overweight girls; ^||^ *p <* 0.05, normal-weight girls vs. obese girls; ^¶^ *p <* 0.05, overweight boys vs. overweight girls; ** *p <* 0.05, overweight boys vs. obese boys; ^††^ *p <* 0.05, overweight girls vs. obese girls; ^‡‡^ *p <* 0.05, obese boys vs. obese girls.

**Table 2:** Boys and girls in multiple linear regression for LVM, LVEDV, LVESV, LVM-indexed height, and LVM-indexed height^2.7^.

Sex	Variable	LVM			LVEDV			LVESV			LVM-Indexed Height			LVM-Indexed Height^2.7^		
		*β*	SE	*p*	*β*	SE	*p*	*β*	SE	*p*	*β*	SE	*p*	*β*	SE	*p*
Boys	Intercept	−8.44	14.86	0.57	13.37	13.08	0.31	1.25	5.28	0.81	13.91	11.16	0.21	33.32	7.66	<0.001
	BMI	2.94	0.39	<0.001	2.29	0.34	<0.001	0.70	0.14	<0.001	2.21	0.29	<0.001	1.35	0.20	<0.001
	Age	1.96	1.82	0.28	1.30	1.61	0.42	0.80	0.65	0.22	−0.20	1.37	0.89	−2.17	0.94	0.022
	SBP	0.27	0.09	0.002	0.14	0.08	0.074	0.05	0.03	0.12	0.15	0.07	0.025	0.03	0.05	0.57
	DBP	−0.29	0.11	0.009	−0.25	0.10	0.012	−0.07	0.04	0.096	−0.21	0.08	0.012	−0.11	0.06	0.046
	VFA	−0.04	0.05	0.36	−0.13	0.04	0.003	−0.04	0.02	0.010	−0.07	0.04	0.060	−0.08	0.02	0.001
Girls	Intercept	−36.34	12.85	0.041	−4.05	11.33	0.72	0.74	4.72	0.88	−2.97	9.64	0.76	19.14	6.49	0.003
	BMI	2.16	0.35	<0.001	1.49	0.31	<0.001	0.41	0.13	0.001	1.82	0.26	<0.001	1.34	0.18	<0.001
	Age	5.05	1.60	0.002	4.46	1.40	0.002	1.18	0.58	0.043	2.13	1.19	0.075	−0.77	0.80	0.34
	SBP	0.07	0.08	0.35	0.08	0.07	0.25	0.02	0.03	0.51	0.02	0.06	0.74	−0.03	0.04	0.42
	DBP	0.04	0.10	0.65	−0.14	0.09	0.11	−0.02	0.03	0.61	0.02	0.07	0.73	0.01	0.05	0.92
	VFA	0.04	0.05	0.37	0.01	0.04	0.85	0.01	0.02	0.70	−0.03	0.03	0.44	−0.09	0.02	<0.001

LVM: Left ventricular mass; LVEDV: left ventricular end-diastolic volume; LVESV: left ventricular end-systolic volume; BMI: body mass index; SBP: systolic blood pressure; DBP: diastolic blood pressure; VFA: visceral fat area.

## Data Availability

The data that support the findings of this study are not publicly available but are available from the corresponding author upon reasonable request.

## Supplementary Materials

The following supporting information can be downloaded at: https://www.mdpi.com/article/10.3390/nu15133066/s1, Table S1: Sex differences in linear regression for left ventricular mass, end-diastolic volume, end-systolic volume, LVM-indexed height, and LVM-indexed height^2.7^; Table S2: Sex-specific Pearson correlations between obesity indicators and cardiac structure parameters; Figure S1: Sex-specific correlations between body mass index (BMI) and left ventricular mass (LVM) indexed height (A, boys; B, girls) presenting a steeper relationship between BMI and LVM-indexed height in boys. Mean ± 95% CI (dashed lines) is shown for each graph; Figure S2: Sex-specific correlations between body mass index (BMI) and left ventricular mass (LVM) indexed height^2.7^ (A, boys; B, girls) presenting a steeper relationship between BMI and LVM height^2.7^ in girls. Mean ± 95% CI (dashed lines) is shown for each graph.
