# Supplementary material for: Sex-Specific Differences in Left Ventricular Mass and Volumes with Body Mass Index among Children Aged 6 to 8: A Cross-Sectional Study in China
Source: Nutrients. 2023 Jul 7;15(13):3066. doi: 10.3390/nu15133066 (PMC10347080; doi:10.3390/nu15133066)
Supplement: Supplementary file 1 [file nutrients-15-03066-s001.zip › nutrients-2427914-supplementary.pdf]

Table S1. Sex differences in linear regression for LVM, LVEDV, LVESV, LVM indexed height, and LVM indexed height$^{2.7}$.

	Boys		Girls	
	β	***P***	***β***	***P***
**LVM(g)**				
BMI(kg/m^2)	2.82	<0.001	2.47	<0.001
Age(years)	4.75	0.030	5.14	0.005
SBP(mmHg)	0.40	<0.001	0.26	<0.001
DBP(mmHg)	-0.04	0.74	0.28	0.004
Waist circumference(cm)	0.94	<0.001	0.90	<0.001
VFA(cm^2)	0.29	<0.001	0.29	<0.001
Fat mass(kg)	1.91	<0.001	1.82	<0.001
**LVEDV(mL)**				
BMI(kg/m^2)	1.50	<0.001	1.53	<0.001
Age(years)	2.99	0.085	4.05	0.007
SBP(mmHg)	0.17	0.013	0.14	0.021
DBP(mmHg)	-0.12	0.19	0.04	0.58
Waist circumference(cm)	0.52	<0.001	0.57	<0.001
VFA(cm^2)	0.13	<0.001	0.18	<0.001
Fat mass(kg)	0.92	<0.001	1.13	<0.001
**LVESV(mL)**				
BMI(kg/m^2)	0.43	<0.001	0.46	<0.001
Age(years)	1.31	0.051	1.13	0.060
SBP(mmHg)	0.06	0.024	0.04	0.061
DBP(mmHg)	-0.02	0.52	0.03	0.32
Waist circumference(cm)	0.14	<0.001	0.17	<0.001
VFA(cm^2)	0.03	<0.001	0.05	<0.001
Fat mass(kg)	0.26	<0.001	0.34	<0.001
**LVM indexed height(g/m)**				
BMI(kg/m^2)	1.82	<0.001	1.66	<0.001
Age(years)	1.66	0.29	1.96	0.14
SBP(mmHg)	0.22	<0.001	0.15	0.004
DBP(mmHg)	-0.06	0.43	0.15	0.026
Waist circumference(cm)	0.57	<0.001	0.57	<0.001
VFA(cm^2)	0.18	<0.001	0.18	<0.001
Fat mass(kg)	1.20	<0.001	1.16	<0.001
**LVM indexed height$^{2.7}$(g/m$^{2.7}$)**				
Body mass index(kg/m^2)	0.78	<0.001	0.79	<0.001
Age(years)	-1.30	0.19	-1.15	0.18
SBP(mmHg)	0.04	0.28	0.03	0.31
DBP(mmHg)	-0.08	0.11	0.03	0.56
Waist circumference(cm)	0.19	<0.001	0.22	<0.001
VFA(cm^2)	0.06	<0.001	0.06	<0.001
Fat mass(kg)	0.45	<0.001	0.46	<0.001

LVM: Left ventricular mass; LVEDV: left ventricular end-diastolic volume; LVESV: left ventricular end-systolic volume; BMI: body mass index; SBP: systolic blood pressure; DBP: diastolic blood pressure; VFA: visceral fat area.

Table S2. Sex-specific Pearson correlations between obesity indicators and cardiac structure parameters.

Variables	Boys					Girls				
	**BMI**	**Fat mass**	**Waist circumference**	**VFA**	**SV**	**BMI**	**Fat mass**	**Waist circumference**	**VFA**	**SV**
LVM	0.55	0.54	0.53	0.45	0.69	0.51	0.51	0.52	0.45	0.69
P	<0.001	<0.001	<0.001	<0.001	<0.001	<0.001	<0.001	<0.001	<0.001	<0.001
LVM indexed height	0.50	0.47	0.45	0.38	0.67	0.48	0.45	0.46	0.38	0.66
P	<0.001	<0.001	<0.001	<0.001	<0.001	<0.001	<0.001	<0.001	<0.001	<0.001
LVM indexed height$^{2.7}$	0.33	0.28	0.23	0.21	0.54	0.35	0.28	0.28	0.21	0.54
P	<0.001	<0.001	<0.001	<0.001	<0.001	<0.001	<0.001	<0.001	<0.001	<0.001
LVEDV	0.37	0.33	0.37	0.25	0.95	0.39	0.39	0.41	0.33	0.94
P	<0.001	<0.001	<0.001	<0.001	<0.001	<0.001	<0.001	<0.001	<0.001	<0.001
LVESV	0.27	0.24	0.26	0.17	0.57	0.29	0.30	0.30	0.25	0.56
P	<0.001	<0.001	<0.001	<0.001	<0.001	<0.001	<0.001	<0.001	<0.001	<0.001

LVM: Left ventricular mass; LVEDV: left ventricular end-diastolic volume; LVESV: left ventricular end-systolic volume; BMI: body mass index; SBP: systolic blood pressure; DBP: diastolic blood pressure; VFA: visceral fat area; SV: stroke volume.

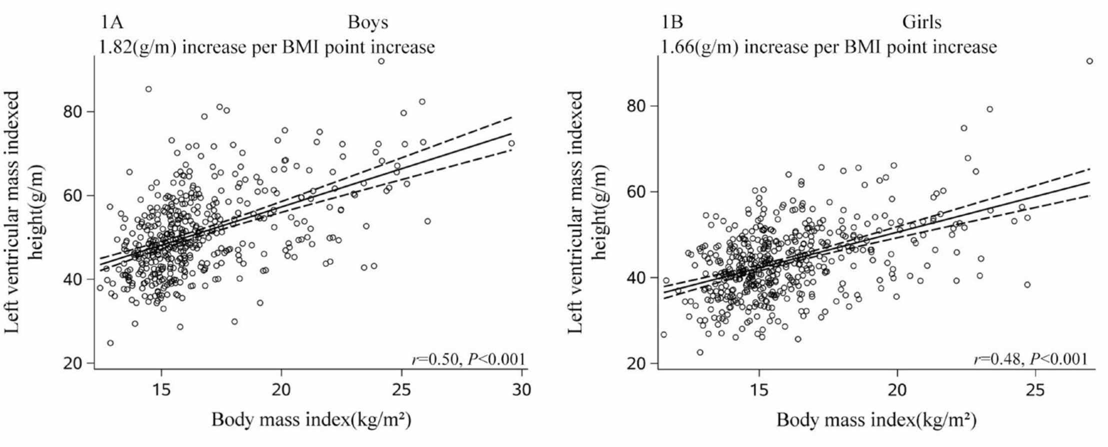

Figure S1. Sex-specific correlations between body mass index (BMI) and left ventricular mass (LVM) indexed height (A, boys; B, girls) presenting a steeper relationship between BMI and LVM indexed height in boys. Mean±95%CI (dashed lines) was shown for each graph.

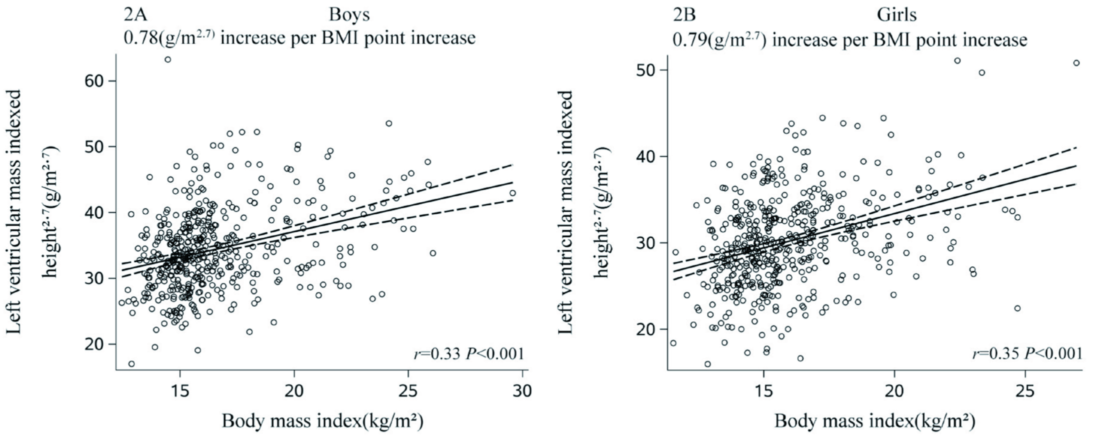

Figure S2. Sex-specific correlations between body mass index (BMI) and left ventricular mass (LVM) indexed height$^{2.7}$(A, boys; B, girls) presenting a steeper relationship between BMI and LVM height$^{2.7}$ in girls. Mean±95%CI (dashed lines) was shown for each graph.
